# Understanding cost drivers and economic potential of two variants of ionic liquid pretreatment for cellulosic biofuel production

**DOI:** 10.1186/1754-6834-7-86

**Published:** 2014-06-07

**Authors:** NVSN Murthy Konda, Jian Shi, Seema Singh, Harvey W Blanch, Blake A Simmons, Daniel Klein-Marcuschamer

**Affiliations:** 1 Joint BioEnergy Institute, 5885 Hollis Street, 94608 Emeryville, CA, USA; 2 Physical Biosciences Division, Lawrence Berkeley National Laboratory, 94720 Berkeley, CA, USA; 3 Biological and Materials Science Center, Sandia National Laboratories, 94551 Livermore, CA, USA; 4 Department of Chemical Engineering, University of California, 94720 Berkeley, CA, USA; 5 Dow Center for Sustainable Engineering Innovation, University of Queensland, St. Lucia, QLD, Australia

**Keywords:** Lignocellulosic biofuels, Ionic liquid pretreatment, Techno-economic analysis, One-pot process, Lignin valorization, Process modeling

## Abstract

**Background:**

Ionic liquid (IL) pretreatment could enable an economically viable route to produce biofuels by providing efficient means to extract sugars and lignin from lignocellulosic biomass. However, to realize this, novel IL-based processes need to be developed in order to minimize the overall production costs and accelerate commercial viability. In this study, two variants of IL-based processes are considered: one based on complete removal of the IL prior to hydrolysis using a water-wash (WW) step and the other based on a “one-pot” (OP) process that does not require IL removal prior to saccharification. Detailed techno-economic analysis (TEA) of these two routes was carried out to understand the cost drivers, economic potential (minimum ethanol selling price, MESP), and relative merits and challenges of each route.

**Results:**

At high biomass loading (50%), both routes exhibited comparable economic performance with an MESP of $6.3/gal. With the possible advances identified (reduced water or acid/base consumption, improved conversion in pretreatment, and lignin valorization), the MESP could be reduced to around $3/gal ($3.2 in the WW route and $2.8 in the OP route).

**Conclusions:**

It was found that, to be competitive at industrial scale, lowered cost of ILs used and higher biomass loadings (50%) are essential for both routes, and in particular for the OP route. Overall, while the economic potential of both routes appears to be comparable at higher biomass loadings, the OP route showed the benefit of lower water consumption at the plant level, an important cost and sustainability consideration for biorefineries.

## Background

Lignocellulosic biomass is the most abundant renewable source of carbon on the planet; thus, it is widely seen as an abundant and sustainable feedstock for the production of non-food-derived biofuels. In addition, lignocellulosic biofuels generally reduce net CO_2_ emissions from the transport sector and thus are considered environmentally benign. The recalcitrance of biomass to depolymerization, however, is one of the key roadblocks for commercial viability of cellulosic biofuels. Pretreatment is essential to *deconstruct* the complex structures present in biomass and to facilitate efficient breakdown of long-chain polysaccharides (specifically cellulose and hemicellulose) into C6 sugars (hexoses) and C5 sugars (pentoses), which can then be readily fermented to biofuels. The development of efficient and economically viable pretreatment methods is thus critical for the advancement of production technologies for cellulosic biofuels. Several pretreatment technologies are being developed to tackle biomass recalcitrance, including pretreatment with acids, ammonia, hot water, or steam [1-3]. More recently, ionic liquid (IL)-based pretreatment methods using a specialized class of ILs have gained attention due to their effectiveness on a range of biomass types and their ability to disrupt lignin and decrystallize cellulose [4], aiding in downstream hydrolysis and fermentation. Furthermore, a recent study [5] at the Advanced Biofuels Process Demonstration Unit (ABPDU) has demonstrated the successful scale-up of IL-based pretreatment from lab scale to small pilot scale without any operational difficulties or loss in performance. This further demonstrates the promise and prospects of IL pretreatment technologies at the industrial scale.

**Figure 1:**
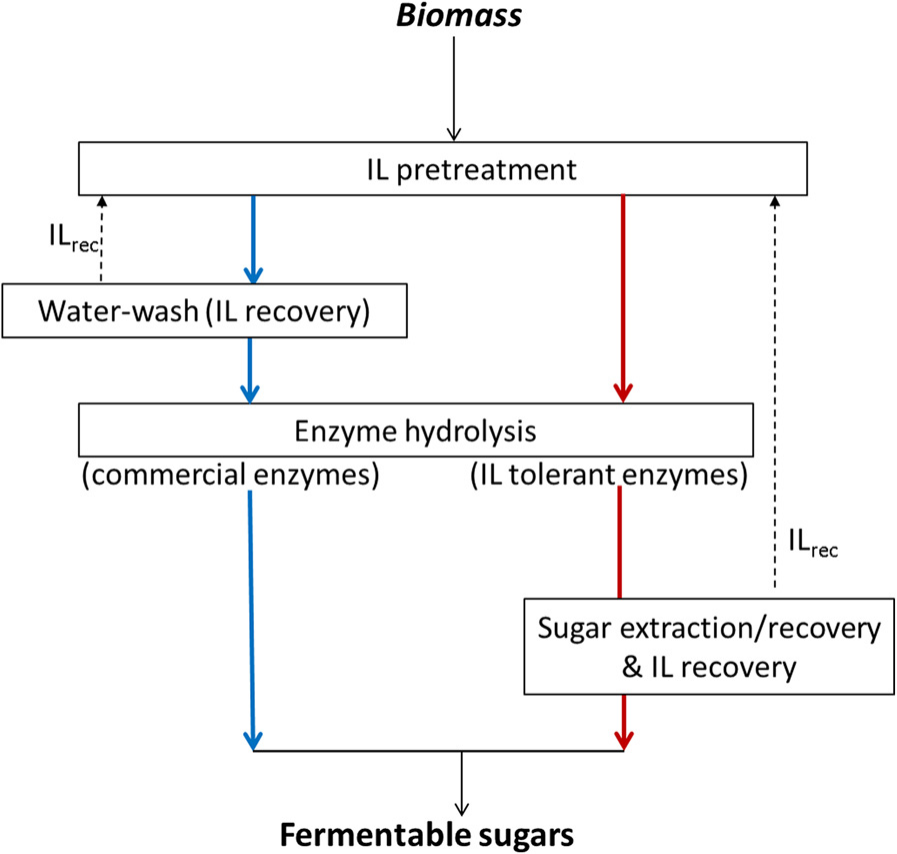
Simplified process flow representation of the water-wash route (blue lines) and the one-pot route (red lines); dashed lines represent IL recycle.

In this study, two variants of IL-based processes are considered: the “water-wash” (WW) and “one-pot” (OP) routes. Simplified process flow representations of both configurations are shown in Figure 1. The primary difference between these two routes is the type of enzyme used for hydrolysis. The WW route uses commercial enzymes that are not tolerant to ILs; thus, the IL must be removed prior to enzymatic hydrolysis. Removal of the IL requires significant amounts of water [6], which may challenge the commercial promise of this technology. On the other hand, the OP route [6] uses a novel enzyme cocktail that is IL-tolerant and facilitates the enzymatic hydrolysis without a separate washing step. As a result of this “consolidated” approach, IL is present during hydrolysis and hence sugars must be extracted from the hydrolyzate prior to fermentation. This is accomplished using liquid-liquid extraction (LLE) techniques, which are shown to recover more than 90% of the sugars from the IL-containing hydrolyzate [7]. After the sugars are extracted, the IL is recovered so that it can be recycled to the pretreatment reactor. Both these routes are further discussed in more detail in the Methods section.

In contrast to other pretreatment technologies, the use of IL-based solvents for biomass dissolution and holocellulose depolymerization is relatively new, and much remains to be learned before an industrial process can be implemented at scale. Recent studies [8-10] have identified some key parameters that can significantly influence the overall process economics of IL-based processes for biorefineries and thus impact the minimum ethanol selling price (MESP, that is, the price of ethanol that results in a zero net present value after discounting cash flows at 10% [11,12]). These parameters include: IL price, IL recovery, and biomass loading (that is, the weight percent of biomass in the pretreatment reactor). For instance, a comprehensive study [10] carried out an extensive set of simulations to quantitatively understand the impact of these IL process parameters on the overall process economics. One of the interesting observations from this study was that high biomass loading (33.3% or higher), low IL price ($2.5/kg or less), and high IL recovery (97% or higher) are needed to ensure an MESP of $5/gal or less. Given that these three factors have already been identified as important cost drivers, our motivation in this study is to understand any other key cost drivers that are specific to the two routes described above. Thus, IL price and recovery are fixed at favorable levels (refer to the *Cost analysis* section). While biomass loading was also known to be a key cost driver, it was further explored in the present study because its impact on the economics of the routes considered was observed to be significantly different. A more comprehensive discussion on the potential of ionic liquids within the context of biorefineries can be found in the recent literature [13-18].

Although the performance of IL-based pretreatment is very promising [13-17], research on how this pretreatment technology would integrate with the processing of lignocellulosic sugars to fuels is limited. This is particularly important because it is at the level of a “biorefinery” where economic viability-determining interactions can be observed. With this in mind, a techno-economic analysis (TEA) of the aforementioned IL-based processes (the WW and OP routes) was undertaken to better understand how this novel pretreatment method can be implemented at industrial scales. An analogous solvent-based process, using γ-valerolactone in place of ionic liquids, has been recently reported [19], and the conclusions of a TEA would thus be similar to those using ionic liquids. We report on the economic performance of each of these processes and focus on understanding the cost drivers and the relative merits and challenges of these two routes.

## Results and discussion

To identify the cost drivers and to assess the economic potential of the two routes discussed above, several scenarios were constructed from the corresponding base case biorefinery models (see the Methods section). As a first step in the analysis, a detailed TEA was performed for the two routes at 10% biomass loading. For these base cases, which represent the translation of laboratory processes to industrial scales, the MESP for the WW route is $8.5/gal while it is $33/gal for the OP process. In order to identify the underlying cost drivers in both of these routes, a more detailed inspection of the cost breakdown was performed.

### Identifying cost drivers in the WW route

**Figure 2:**
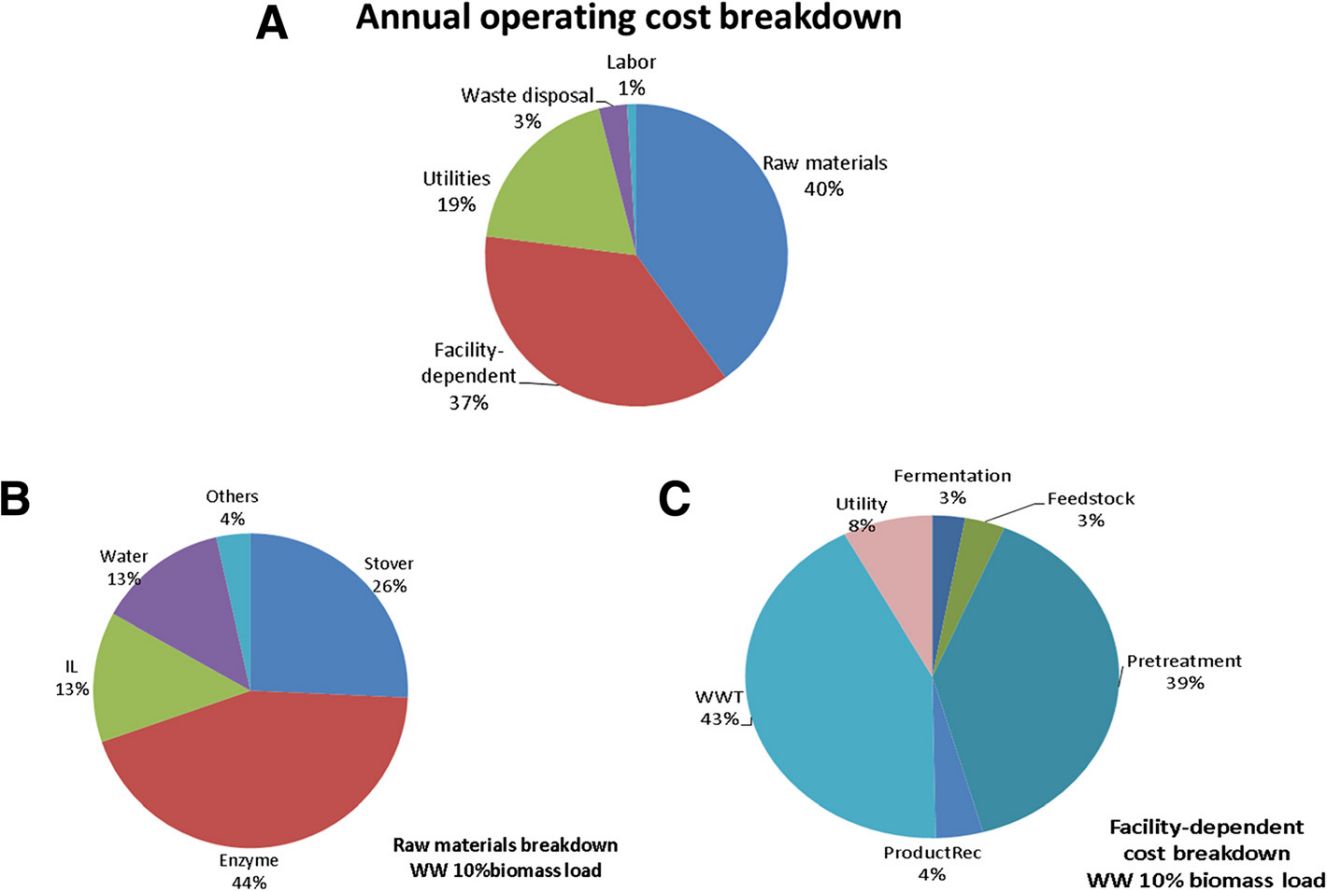
Cost analysis of the water-wash route with 10% biomass loading: AOC (A), raw materials breakdown (B), facility-dependent cost breakdown (C).

A detailed look at the annual operating cost (AOC) breakdown (Figure 2A) revealed that the raw materials and facility-dependent costs (capital depreciation, maintenance, insurance, and overhead) are the most significant cost contributors. Together, they contributed more than three-quarters of the total AOC. Hence, raw materials (Figure 2B) and facility-dependent costs (Figure 2C) were further evaluated in detail. The raw material breakdown revealed that the stover, enzyme, and ILs are significant. Interestingly, the cost contribution of water, which is usually not notable in biofuel processes, appears to be significant (about a sixth of the total raw material expenditure). This is not necessarily unexpected considering the significant amount of water required to recover the IL during the water-wash step. Water loading (i.e., the ratio between water used and biomass present in the water-wash step) is very high (~150) at 10% biomass loading [6]. The effect of high water use impacts more than the raw material cost because the capital required to treat the resulting wastewater must increase accordingly. Indeed, the contribution of the wastewater treatment (WWT) section to the facility-dependent cost was the most significant, followed by the pretreatment section. From this discussion, it is easy to deduce that the water consumption in the WW route must be reduced not only from a sustainability perspective, but also from a process economics perspective, as it affects both the capital and operating expenditures significantly.

### Identifying cost drivers in the OP route

**Figure 3:**
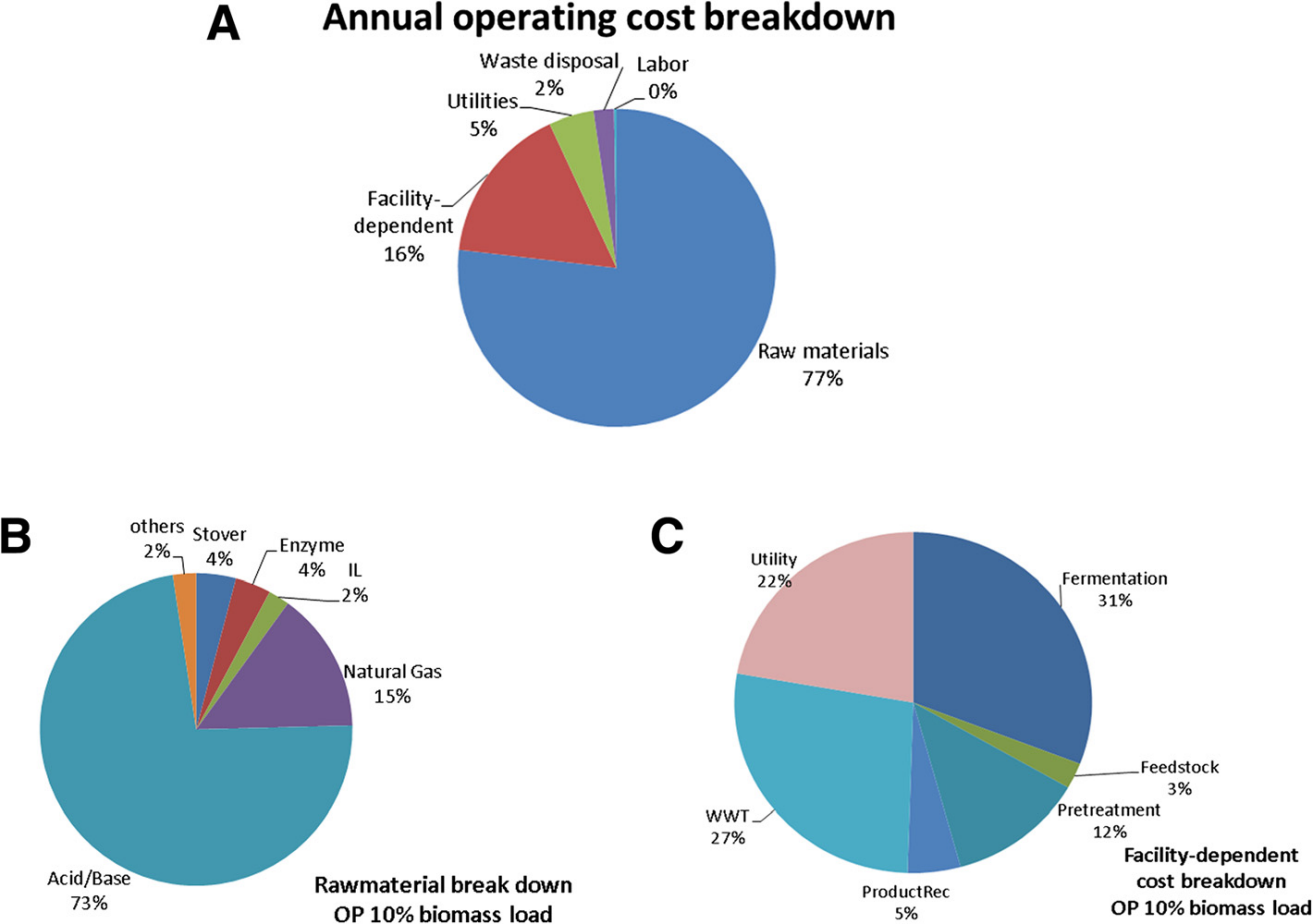
Cost analysis of OP route with 10% biomass loading: AOC (A), raw materials breakdown (B), facility-dependent cost breakdown (C).

From the detailed AOC plot (Figure 3A), raw materials and facility-dependent costs were observed to be significant. Compared to the WW route, the relative cost contribution of raw materials is markedly more significant, followed by facility-dependent costs. As seen in Figure 3B, the contribution of the acid/base needed during sugar extraction and recovery is overwhelming. The total cost of raw materials is about 25 times as much as the feedstock cost itself, while it is four times as much in the WW route. The amount and cost of feedstock in both routes is identical (2,000 dry MT/day, which contributed $45 million/year to the raw materials costs). In absolute terms, the total raw materials cost in the OP route is almost fivefold higher than in the WW route ($1,094 million/year versus $231 million/year), resulting in higher total AOC for the OP-based biorefinery. Because the sugar release levels in both routes are comparable, and hence the overall throughput is comparable (47.6 Mgal/year in the WW route and 45.4 Mgal/year in the OP route), the higher AOC, and not the overall yield, explains the difference in the MESP values. From the capital cost breakdown (Figure 3C), compared to the WW route, the contribution of the WWT section in the OP route is smaller (27% as opposed to 43% in the WW route) due to the elimination of the water-wash step upstream of hydrolysis section. Due to the addition of the sugar extraction/recovery section downstream of the hydrolysis reactor, the capital contribution of the downstream sections (especially fermentation and utility) is more significant in the OP route (facility-related costs for the OP and WW routes is $231 million/year versus $164 million/year, respectively).

### Primary cost drivers in the WW and OP routes

From the above discussion, it is evident that the water consumption is the key cost driver in the case of the WW route whereas the acid/base consumption is the main cost driver in the case of the OP route. In the case of the WW route, the amount of water required and the subsequent capital and operating expenses required for the wastewater treatment contributed 36% of the total AOC. In the OP route, the consumption of acid/base is responsible for 57% of the total AOC. Therefore, before trying to identify any other drivers, it is sensible to evaluate the impact of technological advances with regard to these cost -drivers on the overall conomics of the process.

Incidentally, both the water and acid/base consumption are a function of the biomass loading, with both varying inversely proportional to this variable. In the case of the WW route, the correlation of water use to biomass loading was found experimentally [6], while in case of the OP route, the volume of the acid (and thus base) is proportional to the amount of hydrolyzate entering into the sugar extraction/recovery section. Therefore, biomass loading appears to be a significant factor that can be improved to aid the economics of these processes. This adds a new dimension to the conclusion reached by our previous study [10], in which biomass loading was found to correlate with fresh IL use and therefore a key factor determining the economics of any IL-based process.

### Impact of biomass loading on the WW and OP routes

**Figure 4:**
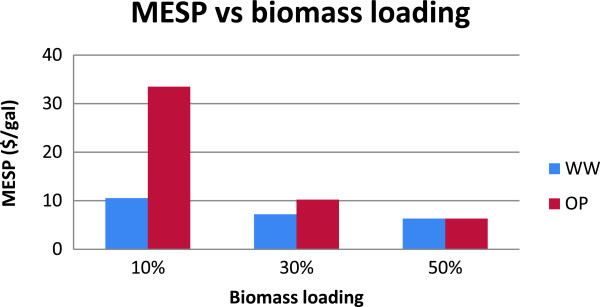
Impact of various levels of biomass loading on the MESP for the WW and OP routes.

Having understood the unfavorable impact of low biomass loading on process economics, we carried out additional scenarios to test the effect of increasing the biomass loading from 10% to 50% (Figure 4). From Figure 4, it was evident that the two technologies behave differently as biomass loading increases. It was interesting to note that the higher biomass loading has a relatively more favorable impact on the OP route compared to the WW route in terms of MESP reduction. This can be qualitatively explained based on the processing differences between these two routes. In the case of the WW route, the water-wash operation (in the pretreatment section) and the WWT section are the only sections that are impacted significantly due to the improved biomass loading and subsequent reduction in the water consumption. In contrast, the impact of higher biomass loading propagates through more sections in the OP-based process. For instance, higher biomass loading reduces the amount of IL required and thus the water consumption in the saccharification section (which is added to maintain an IL concentration of 20% during hydrolysis to ensure effective enzymatic activity). This in turn reduces the volume of the hydrolyzate entering into the sugar extraction/recovery section, hence reducing the need for acid/base and steam required (to maintain columns at 70°C). Overall, the resulting favorable impact, due to high biomass loading, reduced the capital needed in the WWT, fermentation and product recovery (due to less water entering into the system), and utility sections (due to the reduced steam requirement). The complex impact of these benefits acting synergistically in the OP-based process resulted in a more marked impact of biomass loading on the MESP.

**Figure 5:**
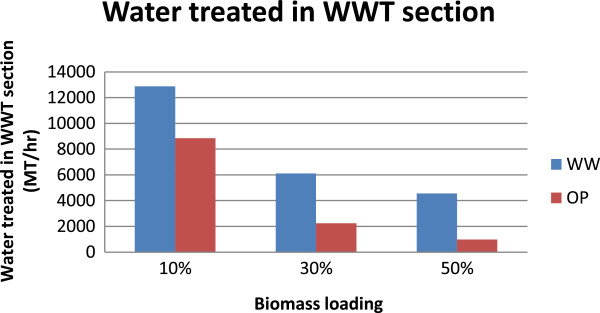
Water treated in wastewater treatment section in the WW and OP routes.

An interesting point to be noted in Figure 4 is that the MESP of both routes is comparable at the 50% biomass loading (~$6.3/gal). In contrast to the WW route, however, the OP-based process uses significantly less water at this biomass loading level [6], in turn producing significantly less wastewater to be treated (Figure 5). Because fresh water is a resource with limited availability (and is likely to become more limited in the future), the OP process would be favored when high biomass loadings are possible. Another interesting observation from Figure 4 is that, as the biomass loading increased, the marginal change/drop in the MESP decelerated, and its value remained above $6/gal even at a biomass loading of 50%. This is an indication that further enhancements are required for either of the routes to compete with current processes (see, for example, [12]).

### Opportunities to further reduce production costs

**Figure 6:**
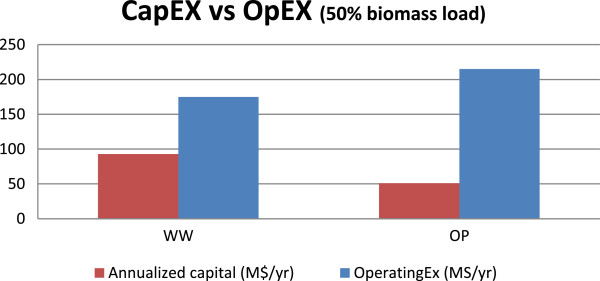
Capital expenditure versus operating expenses in the WW and OP routes.

**Figure 7:**
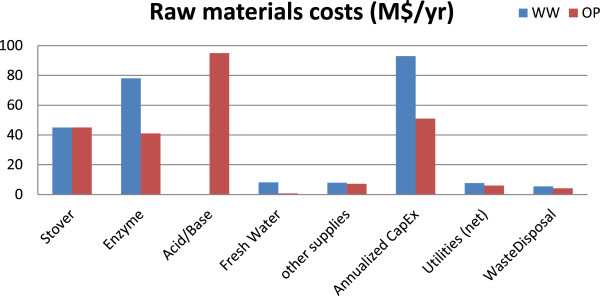
Detailed cost breakdown in the WW and OP routes at 50% biomass loading.

Though the MESP at 50% biomass loading is comparable in both routes, it was interesting to note that their capital and operating costs are very different (Figure 6). The WW route is more capital intensive compared to the OP route. Nonetheless, in both cases the OpEx is the major cost driver (compared to annualized CapEx). A corollary of this observation is that the MESP given in Figure 6 at 50% biomass loading is dependent on the financial assumptions made in the analysis (such as the discount rate and the financing structure).A closer look at the detailed cost split (Figure 7) revealed that the benefits of low enzyme loading in the OP route are outweighed by the significant costs due to acid/base usage. In fact, about 50% of the total raw material cost stems solely from acid/base use in the OP route, which is lower than the 73% of total raw materials cost at 10% biomass loading (Figure 5B) but still very significant. On the other hand, water consumption is still notably higher in the WW-based process compared to that of the OP route, leading to higher WWT capital and resulting in overall higher capital cost. In addition, it can be seen that the utilities and waste disposal costs are comparable in both routes and, more importantly, they are not significant (compared to other components). Hence these two factors were not explored any further as they are unlikely to be the leading determining factors of the overall process economics.

From Figure 7 and as discussed above, even at 50% biomass loading, water usage (and the associated capital investment) remained a key cost factor in the WW route while acid/base consumption in the OP-based process was still significant. In examining the WW route, the water consumption during the water-wash step was varied according to the experimental data [6]; in the OP route, the acid/base utilization rates were taken to be proportional to the hydrolyzate rates (on volumetric basis). All these values were from laboratory-scale experiments, though at industrial scales and with optimized system designs, it may be possible to improve some or all of these factors. For instance, based on pilot-scale experiments [5], water required in the water-wash step was observed to be less compared to the water requirement for the same operation at the lab scale. In addition, with other possible process enhancements at the industrial scale (such as water recycling), water consumption in the WW route can be reduced. Similarly, with further process optimization of the sugar extraction/recovery section, for example, by optimizing operating parameters such as acid/base type and loading, temperature, and pH, the effective acid/base contribution can be significantly reduced (see, for example, [20,21]). These improvements could include the design of an organic solvent (for instance, one with better sugar-extractive capabilities) and the optimization of system conditions such that the amount of organic solvent is reduced, thereby lowering the amount of acid/base chemicals required downstream.

Other significant cost contributors (Figure 7), expectedly, are the enzyme and the stover itself. In this study, enzyme price is fixed at a constant value ($10.14$/kg, [22]). As seen in Figure 7, the total enzyme cost in the case of the OP route is about half of that in the WW route. This difference is due to the fact that the OP route used much less enzyme; nonetheless, this benefit is outweighed by the additional acid/base required in the OP route. Overall, in either route, the cost due to the enzyme is still significant and hence any reduction in enzyme loading (mg enzyme/g glucan) could potentially reduce the overall production costs. The impact of enzyme cost on the overall process economics has been studied in more detail in a previous study [22] and is not discussed further here. While the feedstock price is also fixed (at $58/dry ton, [12]), one way to minimize feedstock cost contribution is by improving the yield. Subsequently, improved conversion during pretreatment could further reduce the MESP.

Based on the above discussion, two additional scenarios were constructed (using the 50% biomass loading cases as the starting point) to evaluate the impact of the possible advances identified in this section. These scenarios are:

• Reduced water or acid/base consumption, with water loading of 20 in the WW route and reduced acid/base consumption in the OP route by 75%.

• Improved conversion (95%) of glucan/xylan to glucose/xylose during pretreatment in both routes.

**Figure 8:**
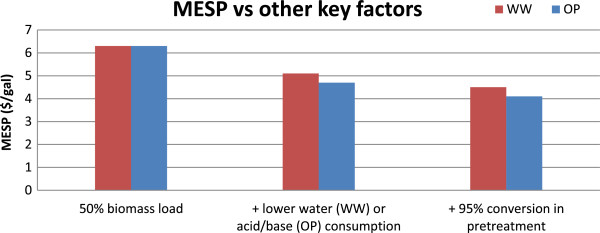
Impact of advances in cost drivers on the MESP in the WW and OP routes.

The MESP values for both these new scenarios, together with the 50% biomass loading scenarios, for both the WW and OP routes are given in Figure 8. The advances in the water and acid/base consumption reduced the MESP from $6.3 to around $5/gal ($5.1 in the WW route and $4.7 in the OP route). Furthermore, improved conversion in the pretreatment section reduced the MESP to $4.5/gal in the WW route and to $4.1/gal in the OP route. Though the MESPs in both the routes are comparable in these scenarios, the OP route seems to have a marginal advantage. In addition, as mentioned earlier, the OP process clearly outperforms the WW route in terms of water usage. The success of the OP route, however, is closely tied with the continued development of novel separation processes that could facilitate inexpensive and efficient sugar extraction and recovery. Within the context of liquid-liquid extraction-based processes, this could mean the use of less expensive acid/base combinations or the use of acids that could be recovered and recycled. Beyond extraction techniques, novel membrane-based processes could offer an alternative avenue of investigation [23,24]. Despite all these perceived advances, however, MESP of the WW and OP routes remained above $4/gal emphasizing the need for further innovation. One such opportunity is discussed in the following section.

### Impact of by-product revenues due to lignin valorization

**Figure 9:**
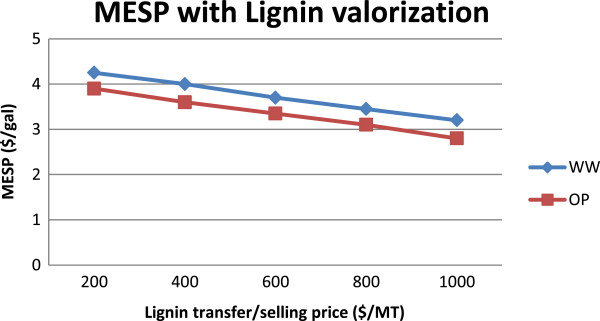
Impact of lignin valorization on the MESP.

In addition to opportunities to reduce production costs, as discussed in the above section, process economics can be improved by increasing revenues (for example, from by-products). For instance, lignin can be spared from being used as a fuel in the co-generation section and instead used to manufacture value-added chemicals [25], creating additional sources of revenue for the biorefinery.Additional scenarios are considered to investigate the impact of lignin valorization on overall process economics by assuming 65% of lignin recovery and a selling price range of $200-1,000/MT (Figure 9) of unprocessed lignin (the “transfer price” to a lignin processing facility). From this analysis, it can be seen that the MESP can be reduced significantly as the lignin selling price increases. For example, at $600/MT, the MESP was around $3.5/gal ($3.7 in the WW route and $3.3 in the OP route), which could be reduced to around $3/gal ($3.2 in the WW route and $2.8 in the OP route) if lignin could be sold at $1,000/MT. This analysis emphasizes the importance of continued research and development on the lignin valorization front.

## Conclusions

A detailed techno-economic analysis (TEA) of two novel routes for IL-based biorefineries was performed. Benchmark economics and the main cost drivers of a water wash (WW) route and a “one-pot” (OP) route to IL pretreatment were identified. Based on the scenarios studied, higher biomass loadings were found to be essential for both routes, though it was a more critical requirement for the OP route. Furthermore, water consumption in the WW route and acid/base consumption in the OP route were observed to be major cost drivers that in turn are correlated with the biomass loading, emphasizing the need to improve this parameter. At high biomass loading (50%), both routes exhibited comparable economic performance (with an MESP of $6.3/gal). The OP route, however, was observed to be more sustainable with regard to water usage. Furthermore, even with the possible advances identified (reduced water or acid/base consumption and improved conversion in pretreatment), the MESP remained above $4/gal ($4.5 and $4.1 in the WW and OP routes, respectively). Further innovation in lignin valorization could reduce the MESP to around $3/gal as the lignin selling price increases to $1,000/MT, highlighting the importance of lignin valorization in the context of future biorefineries. Representative models developed in SuperPro Designer are available to freely download from our wiki [26] for non-commercial usage.

## Methods

### Water-Wash (WW) route

The WW route was designed based on a previous model [10], and the analysis is generally based on previous assumptions as summarized in this section. The process begins with the dissolution of biomass in IL at elevated temperatures (120°C for 0.5 h) with stirring. Water is added to the pretreated biomass in a solid-liquid separation unit to remove most of the IL prior to hydrolysis (the water-wash step). Washing is necessary because commercial enzymes are intolerant to ILs, and hence even a low concentration of IL present during hydrolysis would inhibit enzymatic activity. In order to ensure (near) complete removal of IL, multiple washing stages and a significant amount of water may be required. For instance, a recent study [5] has shown that the water-wash step could involve eight stages to recover most of the IL even with the use of multiple solvents (water and ethanol). After the IL is recovered, the pretreated biomass is diluted with water to a solids loading of 20% (w/w) and the mixture is sent to the hydrolysis reactor. After 10 h of hydrolysis at 50°C, 85% of glucose and xylose sugars are liberated with an enzyme loading of 20 mg/g [12] of polysaccharides (cellulose and hemicellulose together). The hydrolyzate is then sent to the fermentation section to produce ethanol, or any other desired biofuel, which is purified via distillation and molecular sieves.

### One-Pot (OP) route

The OP process is a novel IL pretreatment method that has recently been developed and demonstrated [6] using an IL-tolerant enzyme cocktail JTherm [27]. A brief discussion of this process is given here. Biomass is pretreated with IL at 160°C for 3 h, after which it is diluted with water until the IL concentration is 20% (w/w). The diluted biomass and IL mixture is then hydrolyzed using the JTherm® enzyme cocktail. After 72 h of hydrolysis at 70°C, 81.2% of the glucose and 87.4% of the xylose are liberated with an enzyme loading of 5.75 mg/g of biomass. Solid residue is then separated from the hydrolyzate and, since the IL is not separated prior to hydrolysis, the resulting aqueous hydrolyzate is a mixture of sugars, IL, lignin, and water. Since certain ILs can inhibit microbial activity in the downstream fermentation section, sugars may need to be extracted from this mixture before they can be fermented, which is done using LLE [7] as discussed below.

**Figure 10:**
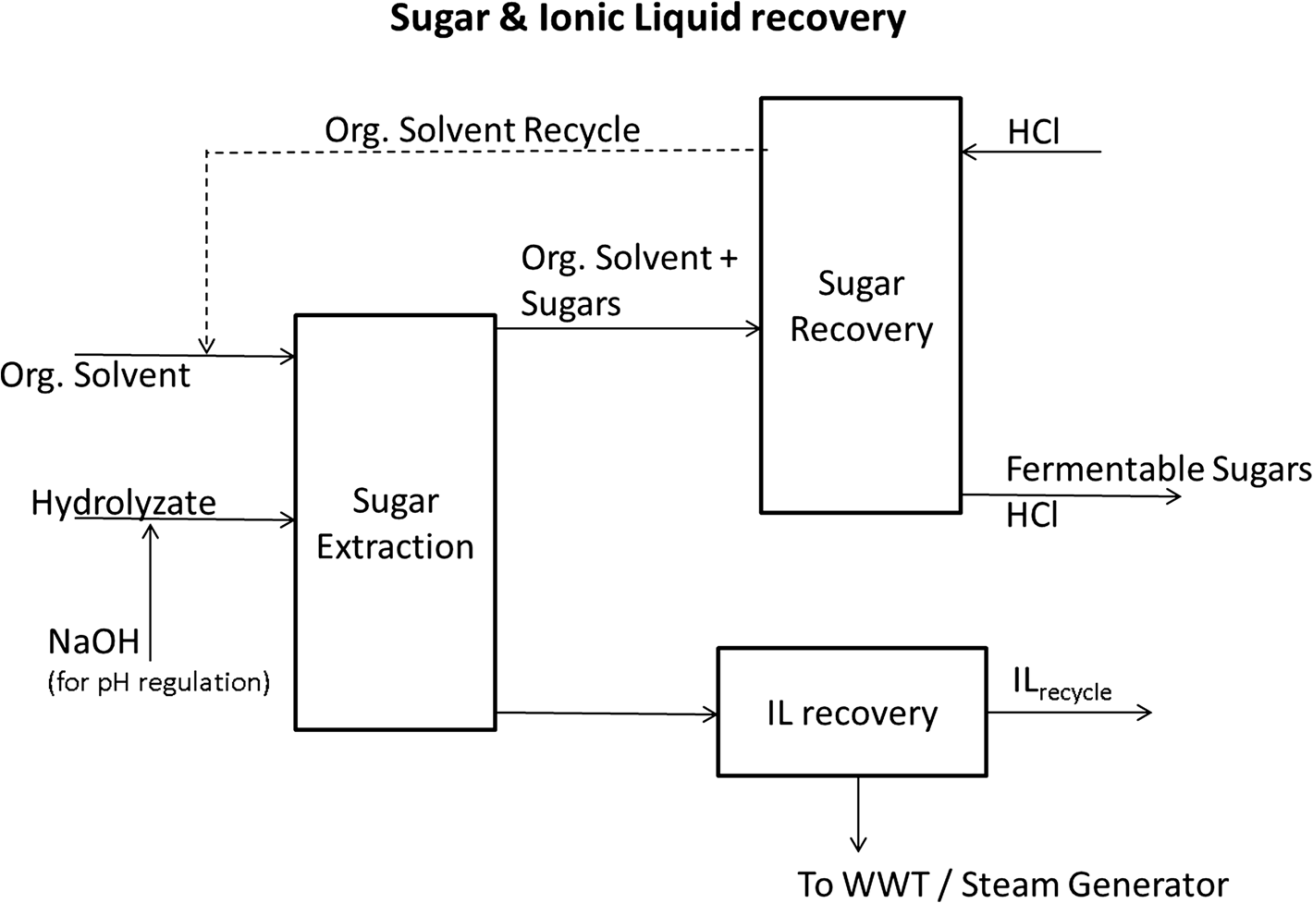
Schematic representation of sugar extraction/recovery section for the OP process using liquid-liquid extraction technique.

A simplified sugar extraction/recovery process flow diagram for the OP process is shown in Figure 10. Since the sugar extraction efficiency of naphthalene-2-boronic acid (N2B) is highest at high levels of pH (~11 to 12), aqueous NaOH (10 M) is added to the hydrolyzate to maintain a favorable pH level. The pH-adjusted hydrolyzate is sent to an extraction column where an equal volume of organic solvent (N2B) is added in countercurrent mode. Sugars are then extracted into the organic phase by forming a boronate complex. Since the complexation reaction reverses under acidic conditions, aqueous HCl (0.5 M, 1:1 v/v) is used to recover the sugars in a second (recovery) column and the organic phase is recycled for reuse. It was assumed that 95% of sugars were extracted in the extraction column followed by 100% sugar recovery in the recovery column [6,7,20,21]. The recovered sugar solution, after neutralization with aqueous NaOH, is sent to the fermentation section to produce ethanol (or any other desired biofuel). The IL from the extraction column bottoms is assumed to be recovered in an IL recovery unit and recycled to the pretreatment reactor, and the remaining mixture (mostly water and lignin) is sent to the wastewater treatment section.

### Base case biorefinery models for WW and OP routes

**Figure 11:**
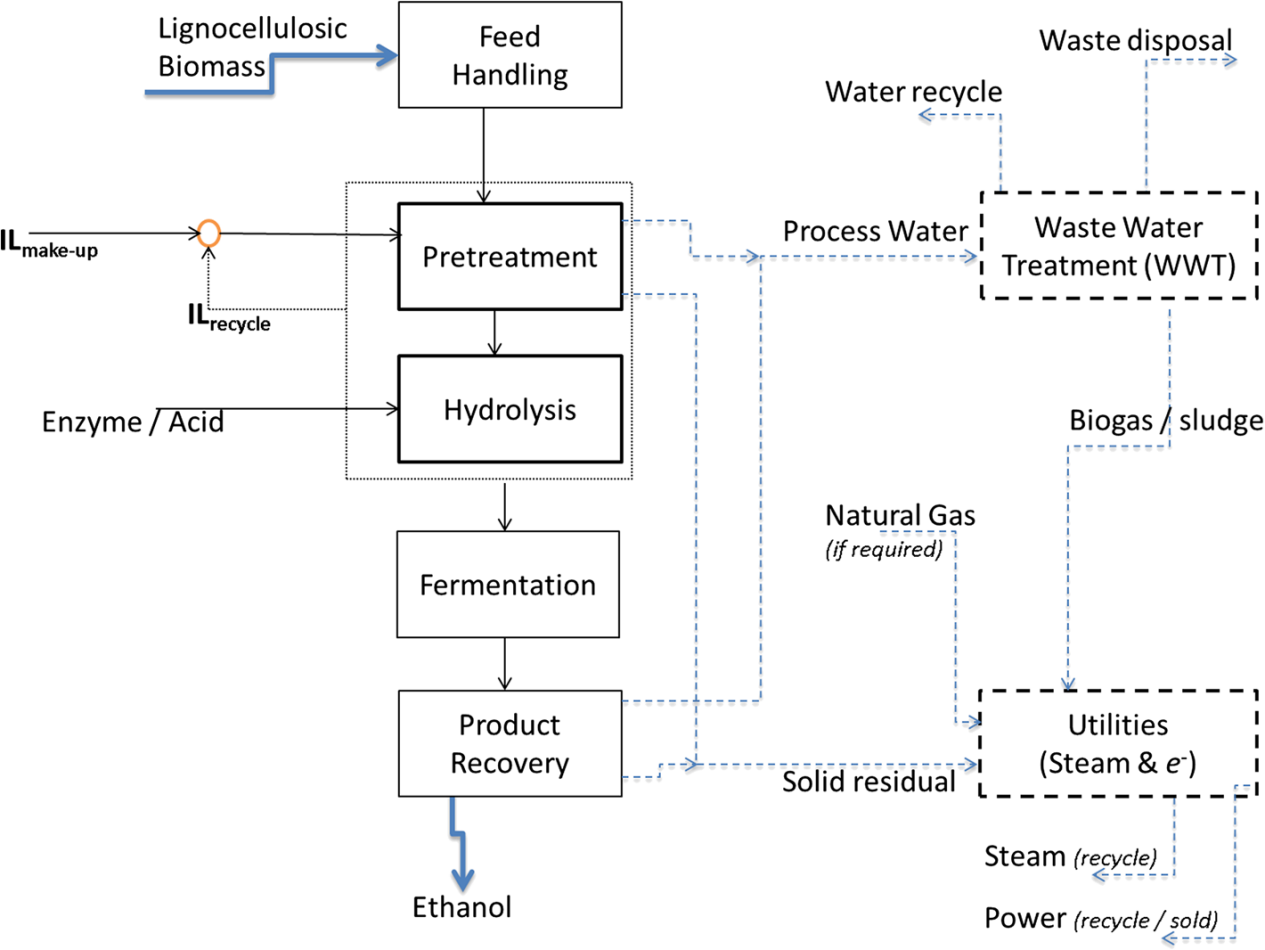
Block flow diagram of the biofuel production pathways modeled.

The two pretreatment routes were modeled, as part of a lignocellulosic ethanol biorefinery, in order to compare the economic performance of each route. A simplified block flow diagram of the biorefinery is shown in Figure 11. Any change(s) in one section (pretreatment in this case/study) could favorably or unfavorably influence other sections. Hence, to gauge the economic performance of different pretreatment options, the whole biorefinery was modeled and TEA was performed over the entire process.

The majority of the process flows and unit operations for the biorefinery (except for the pretreatment section) developed for the base case models for both routes in this study (Figure 11) are based on a recent study by NREL [12] on the production of ethanol from corn stover (with a scale of 2,000 MT/day of dry biomass). Based on the process configuration in Figure 11, ethanol production from lignocellulosic biomass involves five major consecutive steps: feedstock handling, pretreatment, hydrolysis, fermentation, and product recovery. In addition, there are two auxiliary sections: wastewater treatment (WWT) and co-generation (or utilities). While the level of process integration varies from one biorefinery to another, the auxiliary sections (WWT and utility) are typically closely integrated with other sections in the plant. In this study, all the wastewater produced in the process was processed in the WWT section so that it can be reused or discharged as required. Given the biofuel-water nexus, WWT is a key section for the development of future biorefineries while advancing towards “zero-discharge” systems (for example, 100% water recycle). Most of the solid residues from various sections and biogas from the WWT section were sent to the boiler in the co-generation section to produce steam that was used to supply the plant’s steam requirement. The co-generation section was designed such that the plant is self-sufficient with respect to steam. Hence, if the steam produced by burning solid residues and biogas is not sufficient, natural gas was purchased from external sources to meet the overall steam demand of the biorefinery. Excess steam was used in a multistage turbo-generator to produce electricity; this electricity was used for the process needs and any surplus electricity was sold to the grid. The IL/lignin recovery section is modeled as a generalized process with fixed capital cost [10]. The models can be found at our wiki [26], and interested readers can freely download them for non-commercial purposes.

### Cost analysis

For the TEA, the costs of major pieces of equipment, labor, and raw materials were based on previous techno-economic studies [10-12,28,29]. In this study, IL price and recovery, which were already shown to be critical for the economic viability of an IL-based biorefinery [10], were assumed to be constant at favorable levels ($0.75/kg [30] and 99.6% [10,31]) so that the importance of other process parameters specific to WW and OP routes can be studied. The reference year was updated to 2012 and, accordingly, costs were adjusted using the Chemical Engineering Plant Cost Index (CEPCI) and inflation data. The financial assumptions and the economic analysis were taken from previous studies [11,28]. In line with these studies, the results are reported in terms of the minimum ethanol selling price (MESP). Additional details can be found in other studies [12,32].

## Abbreviations

DA: dilute acid; IL: ionic liquid; MESP: minimum ethanol selling price; Mgal: million gallons; OP: one-pot; WW: water-wash.

## Competing interests

The authors declare that they have no competing interests.

## Authors’ contributions

NVSNMK carried out the TEA and drafted the manuscript. NVSNMK and DKM conceived the scope and structure of this study. DKM helped in drafting the manuscript and participated in discussions on the results and analysis. DKM, BAS, HWB, and SS supervised this work and edited the manuscript. JS helped with the IL-based process flows, participated in discussions on the results and analysis, and edited the manuscript. All the authors read and approved the manuscript.
